# Data on chemical-gene interactions and biological categories enriched with genes sensitive to chemical exposures

**DOI:** 10.1016/j.dib.2020.106398

**Published:** 2020-10-09

**Authors:** Alexander Suvorov, Victoria Salemme, Joseph McGaunn, Anthony Poluyanoff, Saira Amir

**Affiliations:** a Department of Environmental Health Sciences, School of Public Health and Health Sciences, University of Massachusetts, 173B-Goessmann, 686 North Pleasant Street, Amherst, MA 01003, USA; b Department of Biosciences, COMSATS University Islamabad, Pakistan

**Keywords:** Toxicity pathway, Adverse outcome pathway, Toxicogenomics, Computational toxicology

## Abstract

A dataset of chemical-gene interactions was created by extracting data from the Comparative Toxicogenomics Database (CTD) with the following filtering criteria: data was extracted only from experiments that used human, rat, or mouse cells/tissues and used high-throughput approaches for gene expression analysis. Genes not present in genomes of all three species were filtered out. The resulting dataset included 591,084 chemical-gene interaction. All chemical compounds in the database were annotated for their major uses. For every gene in the database number of chemical-gene interactions was calculated and used as a metric of gene sensitivity to a variety of chemical exposures. The lists of genes with corresponding numbers of chemical-gene interactions were used in gene-set enrichment analysis (GSEA) to identify potential sensitivity to chemical exposures of molecular pathways in Hallmark, KEGG and Reactome collections. Thus, data presented here represent unbiased and searchable datasets of sensitivity of genes and molecular pathways to a broad range of chemical exposures. As such the data can be used for a diverse range of toxicological and regulatory applications. Approach for the identification of molecular mechanisms sensitive to chemical exposures may inform regulatory toxicology about best toxicity testing strategies. Analysis of sensitivity of genes and molecular pathways to chemical exposures based on these datasets was published in Chemosphere (Suvorov et al., 2021) [1].

## Specifications Table

**Table:** 

Subject	Toxicology
Specific subject area	Computational toxicology
Type of data	Table Graph
How data were acquired	Data on chemical-gene interactions were extracted from the Comparative Toxicogenomics Database (CTD) and processed using MS Excel and Gene Set Enrichment Analysis (GSEA)
Data format	Raw and analysed
Parameters for data collection	Primary source of the data (raw data) for our study is CTD (http://ctdbase.org/). To identify in an unbiased manner molecular mechanisms sensitive to chemical exposures we extracted from the CTD data on chemical-gene interactions coming from high-throughput transcriptomic studies only. Additionally, we restricted our data to only experiments with three organisms (humans, mice and rats) - the major sources of toxicological information to assess risks of toxicity for humans.
Description of data collection	The raw data for this study is the CTD (http://ctdbase.org/). CTD team have extracted from the CTD only data with our filtering criteria. Further, genes not present in genomes of all three species (Mus musculus, Rattus norvegicus and Homo sapience) were filtered out. Chemicals were manually annotated for their major uses. For annotation, search was done in Wikipedia, PubChem and PubMed. The resulting dataset was used to identify genes, molecular pathways and disease categories sensitive to chemical exposures using bioinformatics tools.
Data source location	Primary data source (raw data): Comparative Toxicogenomics Database CTD (http://ctdbase.org/) Institution: North Carolina State University City/Town/Region: Raleigh, NC Country: USA
Data accessibility	Repository name: Mendeley Data Data identification number: 10.17632/65fcympd2j.2 Direct URL to data: https://data.mendeley.com/datasets/65fcympd2j/2
Related research article	A. Suvorov, V. Salemme, J. McGaunn, A. Poluyanoff, M. Teffera, S. Amir, 2020, Unbiased Approach for the Identification of Molecular Mechanisms Sensitive to Chemical Exposures, Chemosphere, https://doi.org/10.1016/j.chemosphere.2020.128362

## Value of the Data

• These data will be useful for the identification of toxicity testing strategies based on the understanding of toxicity pathways and for the understanding of contribution of chemical exposures to different health conditions.
• These data will be beneficial for a broad range of toxicologists, for specialists in assessment of health risks associated with chemical exposures, and for a broad range of medical specialists seeking for the understanding of the role of chemical exposures in a variety of health conditions.
• Our datasets provide information connecting chemical exposures with molecular pathways, as such they can be used to generate new hypotheses and to design new experiments to establish causative links between chemical environment and health.
• These data may be used to develop new priorities in the understanding of the role of chemical exposures in global public health.

## 1 Data Description

GSEA was designed for the analysis of transcriptomic data, where changes in gene expression following treatment go in both directions (increase and decrease in expression). The list of genes is ranked in accordance with expression change values, and usually comparable number of genes have negative and positive values of differential expression. In our study, we ranked all 17,338 genes in accordance with their chemical-gene interaction numbers – figure A. All these values are positive. To make this dataset more suitable for GSEA the same number was subtracted from values of chemical-gene interactions for every gene, to achieve equal negative and positive area under the curve for these values distribution – figure B.

**Dataset: Annotated chemical-gene interactions.xlsx** (Mendeley Data, DOI: 10.17632/65fcympd2j.1 [2])

The data on chemical-gene interactions obtained from high-throughput toxicological genomic experiments with human, mouse, or rat cells and tissues was extracted from the Comparative Toxicogenomic Database (CTD, http://ctdbase.org/) on 08.24.2018. Further, we removed from the database 11,204 genes that are not present in the genome of all 3 species (human, rat and mouse). At the next step chemical compounds were annotated for major uses with information from Wikipedia, PubChem, and PubMed. Based on textual annotation every compound was assigned one to three annotation terms out of the following list: pharmaceutical, recreational drug, research, warfare, endobiotic, agricultural, cosmetics, environment, food components, industrial, and pollutant. The resulting dataset includes 591,084 individual chemical-gene interactions. Each line in the dataset contains information about one unique chemical-gene interaction and includes the following columns: chemical term (chemical name), gene term (gene name), action direction (“+” – expression increase in response to exposure, “−” – expression decrease in response to exposure, “1” – non-specified change in expression in response to exposure), taxonomy (Rattus, Homo or Mus), PMID (PMID number of the original source of data), use1, use2 and use3 (up to 3 annotation terms related to major uses of the chemical compound), use description (textual description of the major uses of the chemical compound).

**Dataset: Number of chemical-gene interactions per gene.xlsx** (Mendeley Data, DOI: 10.17632/65fcympd2j.1 [2])

The dataset created at the previous step was used to determine number of chemical-gene interactions for every gene, including total number as well as number of activating and suppressive chemical-gene interactions. We hypothesize, that the number of chemical gene interactions can be used as a measure of the gene sensitivity to chemical exposures. Each line in the dataset contains information about one unique gene and includes the following columns: gene (gene name), suppressive (total number of suppressive chemical-gene interactions), activating (total number of activating chemical-gene interactions), not specified (total number of chemical-gene interactions with non-specified direction of gene expression change), total (total number of chemical-gene interactions).

**Dataset: Enrichment of molecular pathways with genes sensitive to chemical exposures.xlsx** (Mendeley Data, DOI: 10.17632/65fcympd2j.1 [2])

The list of genes with the total number of chemical-gene interactions for every gene was used as an input for the Gene-Set Enrichment Analysis (GSEA, https://www.gsea-msigdb.org/gsea/index.jsp) against Hallmark, KEGG, and Reactome datasets, to identify molecular pathways highly enriched with genes sensitive to chemical exposures. We suggest, that the normalized enrichment score (NES) for every enriched pathway is a measure of the pathway's sensitivity to chemical exposures. Each line in the dataset contains information about one unique molecular pathway/biological category and includes the following columns: collection of gene sets (Hallmark, KEGG or Reactome), gene set name (name of a pathway or biological category, gene set size (number of genes in the gene set), enrichment score, normalised enrichment score, nominal p-value, and FDR q-value.

## 2 Experimental Design, Materials and Methods

A dataset was created by extracting data on chemical-gene interactions from the CTD [3] on 08.24.2018 using the following filtering criteria. First, data was extracted only from experiments that used high-throughput approaches for gene expression analysis (microarray, RNA-seq). In addition, we selected data only from experiments that used human, rat, or mouse cells or tissues for gene expression analysis in *in vitro* and *in vivo* studies. Further, we removed from the database 11,204 genes that are not present in genome of all 3 species (human, rat and mouse). To identify these genes we first constructed lists of genes for which gene/species combinations in the database are represented by only one or two species, but not all three. Further, using Panther [4], we mapped these lists to genomes of species not represented in gene/species pairs in the database to test if this pair was missing due to the insensitivity of the gene to chemical exposures in a particular species, or if this pair was missing due to the fact that this gene is not present in the genome of the species. The resulting database included 591,084 entries, each representing one chemical-gene interaction (significant change in expression of a gene in response to exposure to a chemical compound) reported in a published study.

At the next step, all chemical compounds in the database were manually annotated to identify their major uses. To obtain this information, a search was conducted using the name of the chemical as the keyword in Wikipedia, PubChem, and PubMed. Based on the obtained descriptive annotation, every compound was categorized in accordance with the following terms: pharmaceutical, recreational drug, research, warfare, endobiotic, agricultural, cosmetics, environment, food components, industrial, and pollutant. The term “pharmaceutical” was used for all drugs, including prescription, over the counter, and traditional medicines. Chemicals that are currently going through preclinical and clinical trials were also categorized as “pharmaceutical”. The term “research” was used for chemicals which are mainly used for research purposes, such as components of molecular biology or analytical chemistry protocols. Some of these chemicals, such as protein kinase inhibitors, for example, have been or are currently tested as candidate pharmaceutical drugs. Given that information about preclinical testing is not always readily available [5], we assigned both “pharmaceutical” and “research” annotation terms to them. The term “recreational drug” was used for compounds currently used as recreational drugs, their major metabolites, and compounds that are not currently used as recreational drugs but which possess these properties, making them potential recreational drugs. The term “warfare” was used for compounds used as chemical weapons. The “endobiotic” annotation term was assigned to molecules synthesized in mammalian organisms as normal components of healthy physiology, such as cholesterol, hormones, bile acids, and others. The term “agricultural” was used for fertilizers and pesticides. The term “cosmetics” was used for a broad range of chemical compounds used in cosmetics and perfumes. The annotation term “environment” was used for oxygen and ozone. The term “food component” was used for a broad range of compounds that can be found in food, such as dietary nutrients, food additives, byproducts of food processing, and others. The term “industrial” was used to annotate a broad range of chemicals used as intermediates or final products in a variety of industrial processes, applications, and final products resulting from industrial process. Finally, the term “pollutant” was used for these chemicals which do not have current uses but are produced by natural processes and human activity such as products of incomplete combustion of organic material, air and water pollutants, toxins produced by algal blooms and similar. For chemicals that fell into multiple categories, we used up to three annotation terms reflecting the most common uses. All authors of the manuscript annotated an equal number of chemicals, and all annotations were then checked by the corresponding author to ensure uniformity of annotation approaches.

For every gene in a dataset, we calculated the total number of chemical-gene interactions. Each individual interaction used for this calculation was one line in the dataset and represented a unique combination of the original study, biological model, and chemical compound. Similarly, for each gene, we calculated the total number of activating (gene expression increases in response to exposure) and suppressive (gene expression decreases in response to exposure) interactions.

**Fig. 1:**
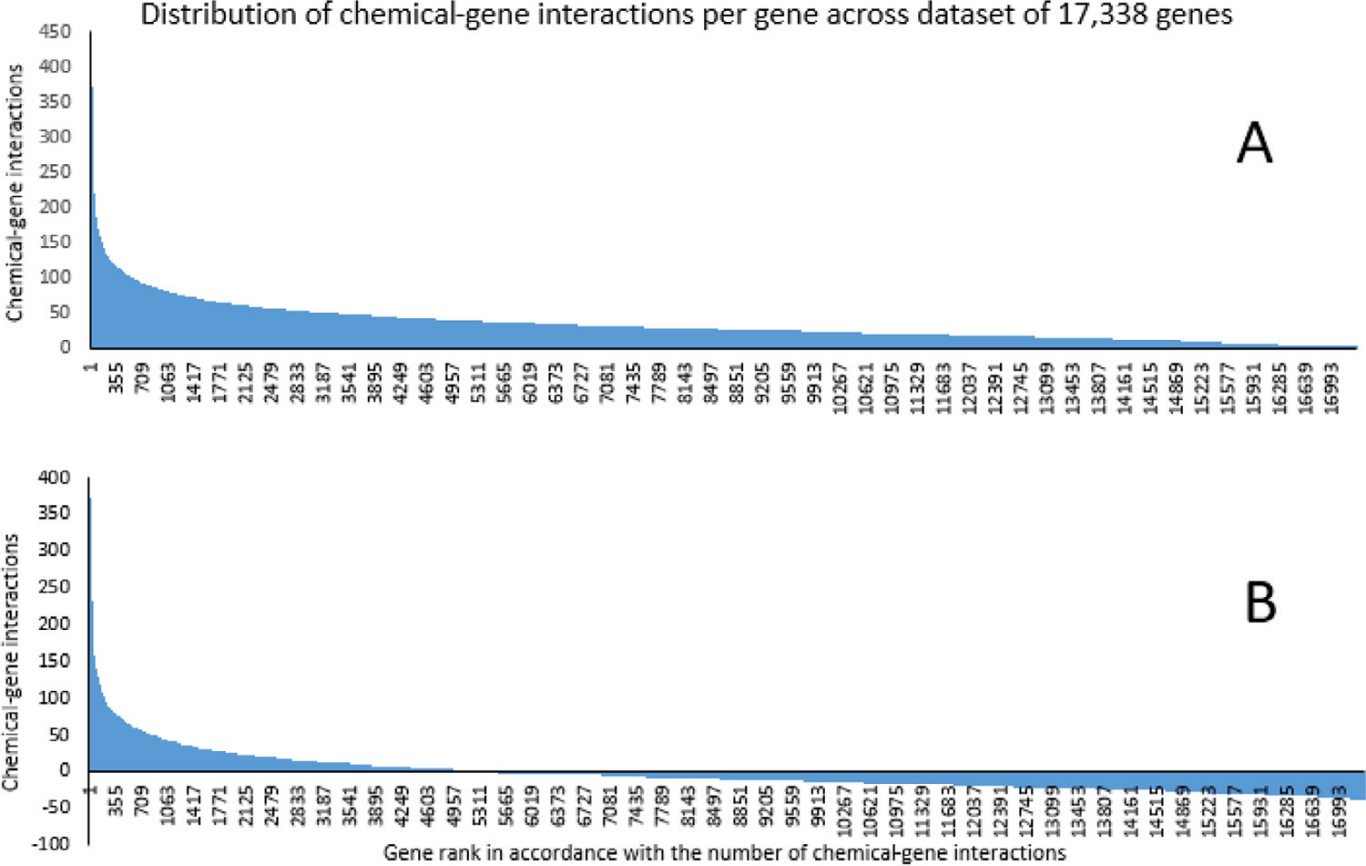
Explanation of data preparation for GSEA analysis.

**Fig. 2:**
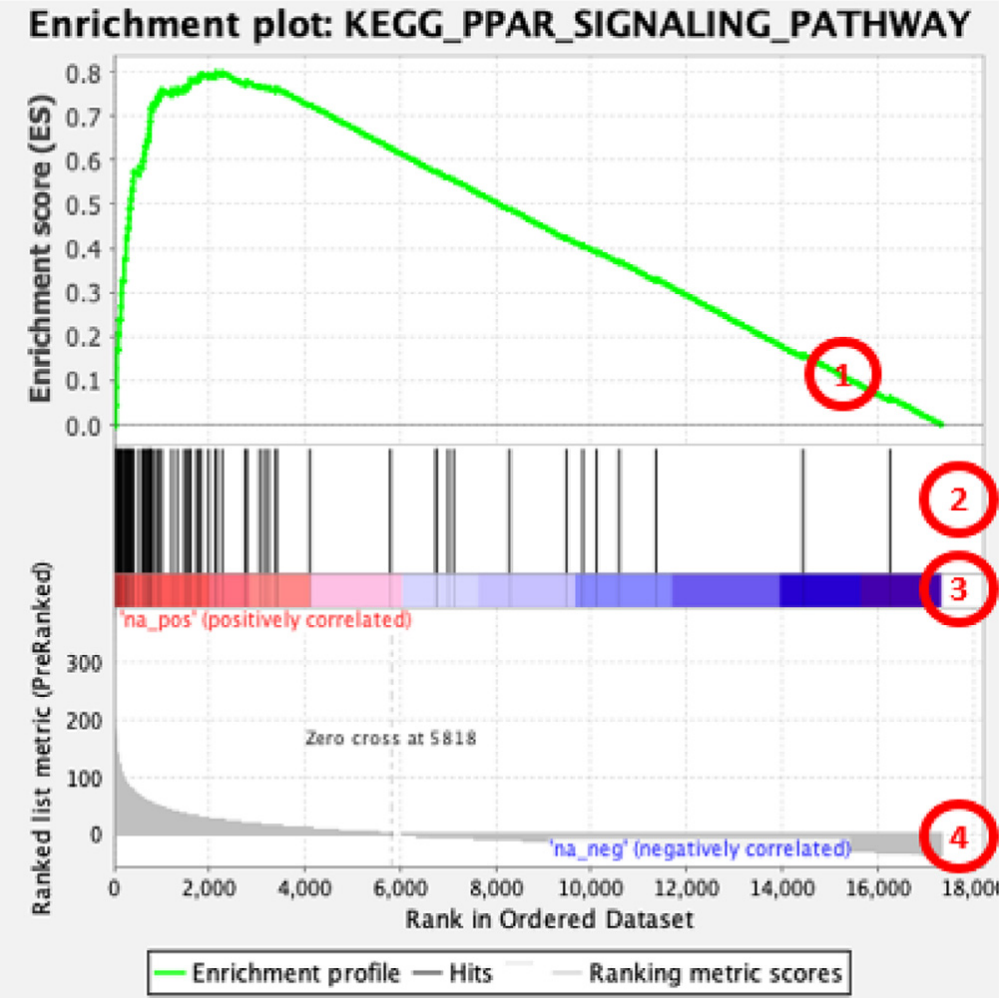
GSEA plot interpretation (based on example of enriched ‘PPAR signalling pathway’ from KEGG collection of datasets): 1 − running enrichment score for the gene set; 2 − vertical lines show where the members of the gene set appear in the ranked list of genes; 3 and 4 – list of genes ranked based on values for chemical-gene interactions from the gene with highest values of interactions (left of the plot) to gene with smallest number of interactions (right of the plot). Number of interactions is shown in the heat-bar (3) and in the bar plot (4). Values of chemical-gene interactions were adjusted to achieve equal negative and positive area under the curve for these values distribution – see Fig. 1.

The lists of all genes with their respective chemical-gene interactions numbers were further used for gene set enrichment analysis (GSEA). This approach is particularly effective for the identification of biologically significant changes in activity of the whole pathway or other biological category, as its enrichment function stems from all, even small, changes in multiple members of a gene set (pathway) [6]. The details of the method and statistical approaches used by GSEA are described elsewhere [7, 8]. In short, an enrichment score (ES) of a gene-set (group of genes from the same biological category) is calculated to reflect the degree to which the gene-set is overrepresented at the top or bottom of the entire ranked list of genes. Thus, GSEA uses as an input the entire available list of genes, rather than a shortlist produced by arbitrary thresholds. The ES is calculated by walking down the gene list and increasing a running-sum statistic when a gene from the gene-set is encountered and decreasing it when genes not in the gene-set are encountered. The final ES is the maximum deviation from zero encountered in the random walk. ES are further normalized to account for the size of the gene-set. Resulting normalized enrichment scores (NES) can be compared across many gene-sets regardless their size. Originally, GSEA was developed to characterize the cumulative shift of all genes in a particular pathway towards an increase or decrease of expression. As such, it was designed for an input in which values of gene expression changes have semi-symmetrical distribution, where comparable numbers of genes are up- and downregulated. To prepare datasets suitable for GSEA, we subtracted the same number from the values of chemical-gene interactions for every gene, to achieve an equal negative and positive area under the curve for these values (Fig. 1). The resulting gene lists were uploaded to GSEA and were analyzed against three independent open-access databases of pathways: Hallmark [9], Reactome [10, 11] and KEGG [12]. The following stringent criteria were used to identify molecular mechanisms most and least sensitive to chemical exposures: normalized enrichment score (NES) ≥ 1.9 or ≤ −1.9 and FDR *q* ≤ 0.05. Interpretation of GSEA plots is shown in Fig. 2. Analysis of sensitivity of genes and molecular pathways based on the datasets presented in the current paper was published recently [1].

## Declaration of Competing Interest

The authors declare that they have no known competing financial interests or personal relationships which have, or could be perceived to have, influenced the work reported in this article.
